# Association between high-risk fertility behaviour and anaemia among urban Indian women (15–49 years)

**DOI:** 10.1186/s12889-024-18254-x

**Published:** 2024-03-09

**Authors:** Sanjay Kumar Pal, Chander Shekhar

**Affiliations:** Department of Fertility & Social Demography, International Institute for Population Sciences, Mumbai-400088, India

**Keywords:** High-risk fertility behaviour, Anaemia, Birth interval, Birth order, Urban

## Abstract

**Background:**

Women in their reproductive age have tremendous health implications that affect their health and well-being. Anaemia is an indicator of inadequate dietary intake and poor health. Maternal malnutrition significantly impacts maternal and child health outcomes, increasing the mother's risk of dying during delivery. High-risk fertility behaviour is a barrier to reducing mother and child mortality. This study aims to examine the level of high-risk fertility behaviour and anaemia among ever-married urban Indian women and also examine the linkages between the both.

**Methods:**

Based on the National Family Health Survey's fifth round of data, the study analyzed 44,225 samples of ever-married urban women. Univariate and bivariate analysis and binary logistic regression have been used for the analysis.

**Results:**

Findings suggested that more than half (55%) of the urban women were anaemic, and about one-fourth (24%) of women had any high-risk fertility behaviour. Furthermore, the results suggest that 20% of women were more vulnerable to anaemia due to high-risk fertility behaviour. For the specific category, 19% and 28% of women were more likely to be anaemic due to single and multiple high-risk fertility. However, after controlling for sociodemographic factors, the findings showed a statistically significant link between high-risk fertility behaviour and anaemia. As a result, 16% of the women were more likely to be anaemic due to high-risk fertility behaviour, and 16% and 24% were more likely to be anaemic due to single and multiple high-risk fertility behaviour, respectively.

**Conclusions:**

The findings exposed that maternal high-risk fertility behaviour is a significant factor in raising the chance of anaemia in ever-married urban women of reproductive age in forms of the short birth interval, advanced maternal age, and advanced maternal age & higher order. Policy and choice-based family planning techniques should be employed to minimize the high-risk fertility behaviour among Indian urban women. This might aid in the reduction of the malnutrition status of their children.

## Background

Malnourishment creates a vicious circle; an individual cannot live a healthy and active life without regular and adequate food [1]. Thus, it is essential to provide adequate food. Due to societal and biological factors, women in Indian society are more susceptible to anaemia throughout their life cycles. Women of reproductive age (15–49) have a variety of stresses that can negatively impact their health and well-being. According to the World Health Organization (WHO), nutritional anaemia is a state in which, regardless of the cause of such insufficiency, the haemoglobin content of blood is lower than the average level. Anaemia is mainly caused by the complex interplay of dietary factors, infectious disease, genetics, and other factors associated with it. Anaemia is a public health problem worldwide and an indicator of poor nutrition and poor health. The burden of anaemia falls mainly in developing countries. The world faces a grave nutrition situation; about 30 percent of women aged 15–49 years suffered from anaemia in 2019, and more than one and a half billion people lack key micronutrients like iron and vitamin A [2]. The nationally represented survey in India documents that more than half (55%) of women (15–49) were found anaemic in the National Family Health Survey (NFHS)-4 (2015–16), which has increased to two-thirds of women (15–49) in NFHS-5 (2019–21) [3, 4].

Anaemia has been linked to several adverse health outcomes, including maternal, perinatal, and neonatal death [5], and it can contribute to high-risk fertility behaviour. Low dietary intake is the main contributor to anaemia in India. Iron, folic acid, vitamin B12, amino acids, proteins, vitamins A and C, and other vitamins from the B complex group, including nicotinic and pantothenic acid, which are also involved in haemoglobin level, are some of the nutrient deficiencies that cause anaemia [6]. Maternal low stature and iron deficiency anaemia, which increase the mother’s risk of dying after delivery, caused at least 20% of maternal deaths [7]. Maternal and children's health are adversely affected by chronic undernutrition, anaemia, and maternal and child mortality, which are linked to high-risk fertility behaviour [8–10]. Maternal age, birth spacing, and parity are characteristics of women's fertility behaviour, which affects both women's and children's health [11, 12]. These parameters, age, parity, and birth spacing, are also called bio-demographic factors. However, the bio-demographic characteristics by themselves do not pose a risk to the mother and her children. Too short birth intervals (less than 24 months) are linked to higher health risks for mothers and newborns [13]. High-risk fertility behaviour among women can be affected by sociodemographic characteristics, residence, religion, level of education, and marital status associated with high-risk fertility behaviour [14, 15].

Women married at 18 years or older have a higher risk of becoming anaemic than those married at 18 years or less [14]. The main factors that affected women's health were early marriage and childbirth, which also account for the current sizeable socio-economic inequality. Higher maternal mortality resulted from inadequate and poor use of healthcare services and widespread anaemia among reproductive-aged women [16]. The prevalence of anaemia among Indian women (15–49) has increased from 55 to 58% during one and half decades (2005–06 to 2019–21). In this period, among rural women, a slower increase was noticed (57.4 to 59%) than among urban women (50.9 to 54%). It indicates that the growth of anaemia among urban women was much higher than their rural counterparts [3, 4]. Urban women face anaemia in India due to their nutritional intake and lifestyle. Urban dwellers' likelihood of developing anaemia was dramatically increased by exposure to a single maternal high-risk behaviour [17]. Presently, Indian urban women are susceptible to being anaemic and have a long history of a higher prevalence of anaemia. With childbearing at an early age, shorter birth intervals, advanced maternal age, and increased susceptibility to frequent children, urban Indian women appear to be at a high risk of anaemia. With this backdrop, there is a need to dig into the study to explore the association between high-risk fertility behaviour and anaemia among urban ever-married women in the reproductive age group (15–49 years) in India.

## Methods

### Data source

This study used the nationally representative microdata from NFHS-5, which was carried out in 2019–21. The survey used a uniform sample design representative at the national, state/union territory, and district levels. A stratified two-stage sampling design was conducted in the 707 districts' rural and urban areas (as of March 31st, 2017). Villages were selected from the sampling frame within each rural stratum using probability proportional to size (PPS), with explicit stratification based on the SC/ST population percentage and female literacy. NFHS-5 covers 636,699 households, with 724,115 eligible women aged 15–49 and 152,752 children aged 6–59 months. The households were selected using a sampling frame created by mapping and listing households in all 707 districts' primary sampling units (PSUs). NFHS-5 used four survey schedules canvassed in local languages utilizing computer-assisted personal interviewing (CAPI) and several data quality assurance procedures to generate accurate and reliable estimates of population, health, and sociodemographic and biomedical indicators development.

The survey provides information on India's population, health and nutrition, and each State/Union territory. The scope of clinical, biochemical testing (CAB) or Anthropometric and Biomarker components has been expanded to include measuring blood pressure and blood glucose levels. NFHS-5 sample has been designed to provide district and higher-level estimates of various indicators covered in the survey. The Biomarker Schedule covered measurements of height, blood pressure, weight, and haemoglobin levels for children and random blood glucose levels for men aged 15–54 years and women aged 15–49 years. For the study of this paper, we have used individual records from the kids’ dataset for the analysis purpose and further restricted to the sample size of 44,225 for our study, considering urban ever-married women aged 15–49 years living with at least one child born to them in the five years prior to the survey date.

### Statistical analysis

Univariate and bivariate analysis has been performed to determine the prevalence of high-risk fertility behaviour and anaemia among urban Indian women. Further, the chi-square test has been used to see the significance level of the output. Binary logistic regression has employed to investigate the relationship between high-risk fertility behaviour and anaemia among urban ever-married women.

Logistic regression is one specific form of a generalized linear model with a logit link function, also known as the log of odds**,** where odds is the probability of success divided by the probability of failure.

A binary logistic regression is usually put into a more compact form as follows:

$$Logit\ \left(pi\right)=ln(\frac{{\text{pi}}}{1-pi})=\alpha +{\upbeta }_{1}\mathrm{x}_{1}+\dots+{\upbeta }_{i}\mathrm{x}_{i}+\dots +{\upbeta }_{k}\mathrm{x}_{k}$$

where $\alpha$ is the intercept, β_1_, β_2_, ………, β_k-1_, βk are the regression coefficients, which indicate the relative effect of a specific explanatory variable on the outcome variable, and $\mathrm{x}_{1}$**,**$\mathrm{x}_{2}$**, ….,** $\mathrm{x}_{k}$, are control variables [30]. Statistical package STATA for windows version 16.0 [31] was performed for all statistical analyses, and weight was used for all the analyses.

For the output of Table 6, the binary logistic regression has been used. For model-I and model-II, the unadjusted model (binary) has been used with the exposure variable any high-risk fertility behaviour (any (i.e., any one of single or multiple) high-risk fertility behaviour vs. no risk) and type of high-risk fertility behaviour (single and multiple high-risk fertility behaviour vs. no risk), respectively. For model-III and model-IV are adjusted models (i.e., including all the covariates) with the exposure variables of any high-risk fertility behaviour and type (single and multiple) of high-risk fertility behaviour, respectively.

### Outcome variable

In order to analyze the level of anaemia of ever-married women, anaemia is considered a dependent variable. The amount of haemoglobin (Hb) per deciliter (dl) of blood, or in grams (g), is used to determine the anaemia level or gm/dl (grams per deciliter). Using WHO-recommended cutoff points for mild, moderate, and severe anaemia is operationalized as a categorical variable. Any anaemia was defined as Hb less than 11.0 g/dl among women; mild anaemia was defined as 10 to 10.9 g/dl, moderate anaemia as 7.0 to 9.9 g/dl, and severe anaemia as less than 7.0 g/dl. The study included both pregnant and non-pregnant women. This study defines anaemia as an outcome variable in a binary form—“not anaemic” (a woman who doesn’t fall under any of the above categories of anaemia) and “anaemic” (a woman who falls under one of the above categories of anaemia).

### Exposure variables

Maternal high-risk fertility behaviour was the exposure of interest in this paper. We adopted the NFHS-3, 2005–2006, the definition of "high-risk fertility behaviour" [18]. Three parameters were considered for the maternal high-risk fertility behaviour: maternal age at the time of birth, birth interval, and birth order to define the high-risk fertility behaviour. Two exposure variables were defined for this analysis: (i) Any high-risk fertility behaviour; (ii) Exposure to different categories of high-risk fertility behaviour (Single high-risk fertility behaviour and multiple high-risk fertility behaviour categories). The presence of any of the following four conditions was termed as a single high-risk fertility behaviour: (i) mothers aged < 18 years at the time of birth; (ii) mothers aged over 34 years at the time of birth; (iii) the latest child born < 24 months after the previous birth; and (iv) latest child of order three or more. The combinations of two or more single conditions are called multiple high-risk fertility behaviour.

### Explanatory variables

Maternal age, maternal education, religion, number of household members, region, caste, wealth index, female height, maternal body mass index (BMI), C-section delivery, and contraceptive usage were the independent factors adopted for the study. These factors were chosen based on the literature review showing the association with women’s status of anaemia in the Indian context.

## Results

The sociodemographic and biomarker characteristics of the sampled women are presented in Table 1. A total of 44,225 samples of urban, ever-married women aged 15–49, with at least one child younger than five years, were considered for the analysis. More than two-thirds (64%) of the women belonged to the age group 25–34, and just above one-fourth (26%) belonged to the age group 15–24, and remaining around 10% to the age group of 35–49 years. Approximately 12% of the women had no education, more than half (52%) had secondary education, and 27% had higher levels of education. About 38% of women had 5–6 household members, and around one-third had more than seven household members. Forty-six percent of women belonged to other backward classes (OBC), 21% to Scheduled Castes (SC), and 28% to others. About 74% of sampled women were Hindu, and approximately 22% were Muslim. Nearly fourteen percent of the women fell in the poor household wealth tercile, and 68% fell in the rich. A broad geographical regionwide distribution suggests that 24% of women were from the South, 21% from the Central, 19% from the West, 17% from the East, and about 16% from the Northern region. More than 9% of women had short stature (< 145 cm). As per the BMI levels, about 22% and 12% of women were overweight and underweight, respectively. The caesarian section performed about 32% of women's deliveries; more than 50% percent had used the modern method, and 13% had used the traditional method in the past. Regarding the women's nutritional status, 55% of women were anaemic.

**Table 1:** Descriptive statistics of sociodemographic and biomarker characteristics of the urban ever-married women in India (*N* = 44,225)

Characteristics	Weighted (Sample)	%
**Maternal Age**		
15–24	11,666	26.4
25–34	28,268	63.9
35–49	4,291	9.7
**Maternal education**		
No education	5,084	11.5
Primary	4,178	9.5
Secondary	22,890	51.8
Higher	12,073	27.3
**Household member**		
1–4 Members	13,154	29.7
5–6 Members	16,774	37.9
7 + Members	14,298	32.3
**Caste**		
Schedule Caste	8,990	21.4
Schedule Tribe	1,908	4.5
Other Backward Class	19,369	46.1
Others	11,767	28.0
Missing	2,191	-
**Religion**		
Hindu	32,685	73.9
Muslim	9,553	21.6
Christian	1,007	2.3
Others	981	2.2
**Wealth Status**		
Poor	6,075	13.7
Middle	7,837	17.7
Rich	30,313	68.5
**Region**		
North	6,996	15.8
Central	9,452	21.4
East	7,659	17.3
North-east	906	2.1
West	8,419	19.0
South	10,793	24.4
**Maternal BMI**		
Underweight	5,461	12.4
Normal	25,341	57.4
Overweight	9,585	21.7
Obese	3,740	8.5
Missing	98	-
**Women's height**		
< 145 cm	4,071	9.2
> 145 cm	40,108	90.8
Missing	46	-
**Delivery Type**		
Vaginal	30,109	68.1
C-section	14,116	31.9
**Contraceptive use**		
Not using	16,038	36.3
Modern methods	22,374	50.6
Traditional methods	5,813	13.1
**Total**	**44,225**	**100.0**

Table 2 presents the levels of anaemia and high-risk fertility behavior (HRFB) among urban ever-married women in India. The results indicate that more than half (55%) of the women were anaemic, and approximately 24% exhibited any HRFB. Specifically, over 20% of women had a single HRFB, while 3.4% had multiple HRFB.

**Table 2:** Level of anaemia and high-risk fertility behaviours among urban ever-married women in India (*N* = 44,225)

Covariates	Weighted sample (n)	(%)
**Anaemia**		
Not anaemic	19,842	44.9
Anaemic	24,383	55.1
**Any high-risk fertility behaviours**		
No risk	33,750	76.3
Any high-risk	10,475	23.7
**Specific high-risk fertility behaviours**		
No risk	33,750	76.3
Single high-risk	8,963	20.3
Multiple high-risk	1,512	3.4
**Total**	**44,225**	**100.0**

**Fig. 1:**
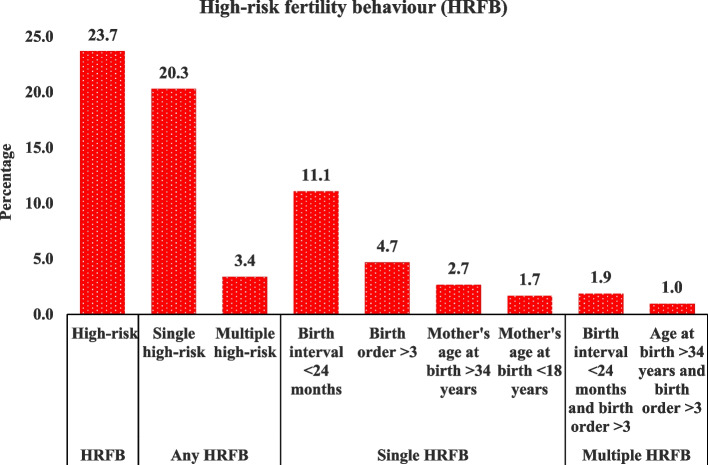
The level (%) of high-risk fertility behaviour (HRFB) (total, any, single and multiple) among ever-married urban Indian women (15–49 years)

Figure 1 shows the high-risk fertility behaviour (HRFB) level among urban ever-married women aged 15–49 in India. A little less than one-quarter (24%) of the women were exposed to any HRFB, 20.3% showed a single, and 3.4% showed multiple high-risk fertility behaviour. For the particular categories of high-risk behaviour, it is clear that a birth interval < 24 months (11.1%), followed by a birth order of more than three (4.7%), was the most common in the single HRFB category. On the other hand, birth interval less than 24 months and birth orders greater than three was the most common multiple HRFB (1.9%), followed by the mother's age at birth greater than 34 years and birth order greater than three (1%).

Table 3 shows the level of high-risk fertility behaviour (HRFB) with sociodemographic characteristics among urban ever-married women. The prevalence of any HRFB (59.5%), single HRFB (42.7%), and multiple HRFB (16.8%) were higher in women aged 35–49 years than the other age groups. Women with no education, household members seven and above, Scheduled Tribes (except for single HRFB), Muslim, poor wealth status, central region, underweight, women with short stature, delivery by vaginal, and using traditional contraceptive methods have a higher prevalence of any, single and multiple HRFB.

**Table 3:** High-risk fertility behaviour with background characteristics among urban ever-married women aged 15–49 years, NFHS-5 (2019–21), India

Characteristics	Any risk	Single risk	Multiple risk
**Maternal Age**			
15–24	21.8	20.8	1.0
25–34	19.1	16.7	2.4
35–49	59.5	42.7	16.8
**Maternal education**			
No education	46.9	33.8	13.1
Primary	35.2	28.8	6.4
Secondary	22.4	20.1	2.3
Higher	12.4	11.9	0.5
**Household member**			
1–4 Members	16.7	16.0	0.7
5–6 Members	24.3	21.1	3.3
7 + Members	29.4	23.3	6.1
**Caste**			
Schedule Caste	26.2	22.7	3.6
Schedule Tribe	26.6	21.9	4.6
Other Backward Class	23.1	20.0	3.1
Others	22.9	19.3	3.6
**Religion**			
Hindu	21.3	18.7	2.6
Muslim	32.6	26.1	6.6
Christian	23.4	21.0	2.4
Others	17.8	16.5	1.3
**Wealth Status**			
Poor	39.2	29.5	9.8
Middle	29.7	25.4	4.4
Rich	19.0	17.1	1.9
**Region**			
North	22.7	19.9	2.9
Central	27.6	22.6	5.0
East	26.9	22.3	4.5
North-east	21.7	17.7	4.0
West	22.7	19.3	3.4
South	19.6	18.0	1.6
**Maternal BMI**			
Underweight	24.5	20.7	3.9
Normal	24.1	20.7	3.4
Overweight	22.4	19.2	3.2
Obese	23.0	19.4	3.7
**Women's height**			
< 145 cm	30.3	24.9	5.4
> 145 cm	22.9	19.7	3.2
**Delivery Type**			
Vaginal	27.3	22.8	4.5
C-section	16.1	14.9	1.2
**Contraceptive use**			
Not using	22.0	18.9	3.1
Modern methods	24.6	21.2	3.4
Traditional methods	25.1	20.6	4.5
**Total**	**23.7**	**20.3**	**3.4**

Table 4 gives the prevalence of anaemia among women by background characteristics. The results depict that more than 58% and 60% of women were found anaemic in the group of single and multiple high-risk fertility behaviour, respectively. About 59% of women aged 15–24 was anaemic, and 52% aged 35–49 were anaemic. Maternal education showed an inverse relationship with the mother's anaemia level except for primary education; as the education level increases, the anaemia level decreases. It is as high as more than 60% of primarily educated women were found anaemic. The number of household members has a positive relationship with anaemia, which showed that women living with a higher number of household members were more anaemia in proportion. A higher proportion of women who belonged to Scheduled Tribes (60%) were anaemic compared with other caste groups. Going by religion, the results suggested that Hindu women were relatively more anaemic (57%) than Muslim (52%) and Christian women (44%). Nearly two-thirds (64%) of women of poor household wealth status were anaemic, and 52% of women of rich household wealth status were anaemic too. The level of anaemia was the highest (67%) in the East region, including the states of Bihar, Jharkhand, Odisha, and West Bengal, and it was the lowest (46%) in the South region, which includes Andaman & Nicobar Islands, Andhra Pradesh, Karnataka, Kerala, Lakshadweep, Puducherry, Tamil Nadu, and Telangana. Underweight and short-stature women were found with a higher level (about 65% & 60%) of anaemia than other counterparts. In the case of contraceptive behaviour, women who were currently using the traditional methods had a higher level (59%) of anaemia than other methods users.

**Table 4:** Prevalence of anaemia among urban ever-married women with background characteristics, NFHS-5 (2019–21), India

Characteristics	Not Anaemic	Anaemic	*p*-value
**High-risk fertility behaviour**			
No risk	46.0	54.1	< 0.001
Single high-risk	41.6	58.4	
Multiple high-risk	39.8	60.2	
**Maternal Age**			< 0.001
15–24	41.4	58.6	
25–34	45.8	54.2	
35–49	48.0	52.1	
**Maternal education**			< 0.001
No education	40.1	59.9	
Primary	39.0	61.1	
Secondary	43.5	56.5	
Higher	51.5	48.5	
**Household member**			0.001
1–4 Members	46.2	53.8	
5–6 Members	45.4	54.6	
7 + Members	43.0	57.0	
**Caste**			< 0.001
Schedule Caste	40.0	60.0	
Schedule Tribe	43.6	56.4	
Other Backward Class	46.8	53.2	
Others	45.8	54.3	
**Religion**			< 0.001
Hindu	43.6	56.5	
Muslim	48.4	51.6	
Christian	55.9	44.2	
Others	43.0	57.1	
**Wealth Status**			< 0.001
Poor	36.0	64.0	
Middle	40.3	59.7	
Rich	47.8	52.2	
**Region**			< 0.001
North	43.9	56.1	
Central	45.1	54.9	
East	32.6	67.4	
North-east	45.1	54.9	
West	44.8	55.2	
South	54.0	46.0	
**Maternal BMI**			< 0.001
Underweight	35.5	64.6	
Normal	44.6	55.5	
Overweight	48.9	51.1	
Obese	50.4	49.6	
**Women's height**			< 0.001
< 145 cm	39.9	60.1	
> 145 cm	45.4	54.6	
**Delivery Type**			0.001
Vaginal	43.7	56.3	
C-section	47.4	52.6	
**Contraceptive use**			< 0.001
Not using	45.9	54.1	
Modern methods	45.1	54.9	
Traditional methods	41.0	59.0	
**Total**	**44.9**	**55.1**	

Table 5 shows the prevalence of anaemia in the different forms of high-risk fertility behaviour (HRFB) in given sociodemographic characteristics. The results reveal that younger women (15–24 years) had a higher level of anaemia in any group of risk than women aged 25 years and above. The level of anaemia was higher (about 69%) among younger women with multiple HRFB. Primarily educated women with single and multiple HRFB were found to have the highest (62.4 & 62.9%) level of anaemia, whereas higher educated women with single high-risk fertility behaviour had the lowest level of anaemia (about 50%). A higher level of anaemia (more than 60%), whether women had single or multiple HRFB, was found among women who belonged to households with seven and more members. Schedule caste women with single high-risk fertility behavior were found to have a high level of anaemia, and also around 68% of Schedule Tribe women with multiple HRFB were anaemic. Hindu women with multiple HRFB had higher (66%) levels of anaemia than women following other religions. Household wealth status showed an inverse relationship with the level of anaemia irrespective of HRFB categories.

**Table 5:** Prevalence of anaemia in the forms of high-risk fertility behaviour among urban ever-married Indian women with background characteristics

Characteristics	Prevalence of anaemia		
	**No risk**	**Single risk**	**Multiple risk**
**Maternal Age**			
15–24	57.0	64.0	68.7
25–34	53.1	58.5	59.3
35–49	50.3	50.7	59.7
***p-value***	**< *0.001***	**< *0.001***	***0.001***
**Maternal education**			
No education	58.5	62.0	60.5
Primary	60.3	62.4	62.9
Secondary	55.9	58.7	58.5
Higher	48.3	49.8	59.6
***p-value***	**< *0.001***	**< *0.001***	***0.102***
**Household member**		0.2	
1–4 Members	53.3	56.4	53.8
5–6 Members	53.5	57.7	60.3
7 + Members	55.5	60.4	60.8
***p-value***	***0.052***	***0.152***	***0.180***
**Caste**			
Schedule Caste	59.1	63.0	60.8
Schedule Tribe	54.6	60.0	67.6
Other Backward Class	51.8	57.7	59.5
Others	53.6	55.6	61.3
***p-value***	**< *0.001***	**< *0.001***	***0.385***
**Religion**			
Hindu	55.3	60.0	66.2
Muslim	50.1	55.3	52.5
Christian	43.5	45.2	55.4
Others	56.4	61.1	48.0
***p-value***	**< *0.001***	**< *0.001***	**< *0.001***
**Wealth Status**			
Poor	63.8	64.3	64.7
Middle	59.0	61.3	61.9
Rich	51.5	55.2	54.6
***p-value***	**< *0.001***	**< *0.001***	**< *0.001***
**Region**			
North	55.1	58.8	61.4
Central	53.8	58.3	56.8
East	66.9	69.5	64.6
North-east	56.0	50.5	52.2
West	54.0	58.1	65.1
South	45.1	49.3	53.3
***p-value***	**< *0.001***	**< *0.001***	***0.002***
**Maternal BMI**			
Underweight	63.1	68.6	70.8
Normal	54.5	58.5	57.9
Overweight	49.6	55.0	63.8
Obese	49.5	50.1	49.0
***p-value***	**< *0.001***	**< *0.001***	**< *0.001***
**Women's height**			
< 145 cm	59.1	60.7	70.7
> 145 cm	53.5	58.1	58.2
***p-value***	**< *0.001***	***0.002***	***0.110***
**Delivery Type**			
Vaginal	54.8	60.0	62.0
C-section	52.7	53.0	45.9
***p-value***	***0.159***	***0.008***	***0.002***
**Contraceptive use**		0.1	
Not using	52.6	59.6	58.4
Modern methods	54.1	56.8	61.1
Traditional methods	58.2	61.5	60.9
***p-value***	**< *0.001***	***0.126***	***0.551***
**Total**	**54.1**	**58.4**	**60.2**

Furthermore, in the case of regions, the Eastern region women with single HRFB had higher levels (69.5%) of anaemia than women staying in the other regions, and the Western region women with multiple HRFB had higher levels (65%) of anaemia than women in the others region. Underweight women with single and multiple HRFB had the highest level (about 69% and 71%) of anaemia than women of normal weight. Short-stature women with multiple HRFB categories were more in proportion to be anaemic (about 71%) than women with normal or taller height. Women who had vaginal delivery were more anaemic than those who underwent C-sections to deliver the last birth. Women with single HRFB and who had vaginal delivery were seven percentage points more anaemic than those who had C-sections delivery. Similarly, women with multiple high-risk fertility behaviour and who had vaginal delivery for their last birth were about 16 percent more anaemic than women who had undergone C-sections. Finally, women using traditional methods of contraception with either single or multiple high-risk fertility behaviour showed a higher proportion of anaemic state than those women who adopted modern methods.

All types of high-risk fertility behavior considered in this study showed a statistically significant association with the status of anaemia among urban married women in India. The results imply that women exposed to any high-risk fertility behaviour (either single or multiple) were 20% more likely to be at risk of anaemic than those exposed to none of the high-risk fertility behaviours. Results specify that women exposed to a single high-risk fertility behaviour were 19% more likely to be at risk of anaemic, whereas those exposed to multiple high-risk fertility behaviour were 28% more likely to be at risk of anaemic than women with no high-risk fertility behaviours. Nevertheless, after controlling the sociodemographic variability, the single and multiple high-risk fertility behaviours alone can make women more likely to be at risk of anaemic, 16% and 24%, respectively, than women in none of the high-risk fertility behaviour categories (Table 6).

**Table 6:** Results of unadjusted and adjusted odds ratio of different forms of high-risk fertility behaviour and anaemia among urban ever-married women (15–49 years), NFHS-5 (2019–21), India

Characteristics	Unadjusted OR [95% CI]		Adjusted OR [95% CI]	
	**Model I**	**Model II**	**Model III**	**Model IV**
**Any high-risk fertility behaviour**				
No risk®				
Any high-risk fertility behaviour	1.20***(1.158 1.253)		1.16***(1.115 1.219)	
**Type of high-risk fertility behaviour**				
No risk®				
Single high-risk fertility behaviour		1.19*** (1.144 1.243)		1.16***(1.106–1.211)
Multiple high-risk fertility behaviour		1.28***(1.165–1.403)		1.24*** (1.118 1.377)
**Maternal Age**				
15–24®				
25–34			0.96*(0.92 1.001)	0.959**(0.919 1.00)
35–49			0.865***(0.807 0.928)	0.86***(0.801 0.923)
**Maternal education**				
No education®				
Primary			1.07*(0.99 1.156)	1.072*(0.992 1.158)
Secondary			1.005 (0.946 1.068)	1.008 (0.948 1.072)
Higher			0.838***(0.781 0.9)	0.841***(0.783 0.903)
**Caste**				
Schedule Caste®				
Schedule Tribe			0.906**(0.826 0.993)	0.905**(0.826 0.992)
Other Backward Class			0.924***(0.881 0.969)	0.924***(0.881 0.969)
Others			0.892***(0.846 0.941)	0.892***(0.845 0.941)
**Religion**				
Hindu®				
Muslim			0.78***(0.745 0.817)	0.78***(0.745 0.817)
Christian			0.812***(0.719 0.918)	0.812***(0.719 0.918)
Others			1.019 (0.902 1.152)	1.02 (0.903 1.152)
**Wealth Status**				
Poor®				
Middle			0.901***(0.861 0.944)	0.902***(0.861 0.945)
Rich			0.824***(0.782 0.867)	0.825***(0.783 0.868)
**Region**				
North®				
Central			0.921***(0.869 0.976)	0.921***(0.869 0.975)
East			1.46***(1.368 1.559)	1.46***(1.368 1.558)
North-east			0.886 (0.767 1.024)	0.886 (0.767 1.024)
West			0.947*(0.893 1.006)	0.947*(0.892 1.005)
South			0.687***(0.647 0.729)	0.687***(0.647 0.729)
**Maternal BMI**				
Underweight®				
Normal			0.717***(0.678 0.758)	0.717***(0.678 0.759)
Overweight			0.67***(0.628 0.714)	0.67***(0.628 0.714)
Obese			0.676***(0.624 0.732)	0.676***(0.624 0.732)
**Women's height**				
< 145 cm®				
> 145 cm			0.924***(0.871 0.98)	0.924***(0.871 0.981)
**Delivery Type**				
Vaginal®				
C-section			1.01 (0.971 1.05)	1.01 (0.971 1.051)
**Contraceptive use**				
Not using®				
Modern methods			1.044**(1.005 1.085)	1.044**(1.005 1.085)
Traditional methods			1.085***(1.025 1.148)	1.085***(1.025 1.148)

^*^p < 0.05

^**^p < 0.01

^***^p < 0.001

## Discussion

The assessment in this paper reveals that around 24% of urban ever-married women experienced at least one high-risk birth, with 20.3% of women experiencing a single high-risk fertility and about 3.4% experiencing multiple high-risk fertility behaviour. Findings also indicate that more than 55% of ever-married urban Indian women were anaemic. Women with higher education (48.5%), who followed the Christian faith (44%), and who were from the South region (46%) had the lowest levels of anaemia. Underweight women (70.8%), short in stature (70.7%), had children between the ages of 15–24 years (68.7%), and belonged to a Scheduled Tribe (67.6%) with multiple high-risk fertility behaviour were the highest to be anaemic across sociodemographic characteristics of women. This study demonstrates that the proportion of women in anaemic state rises as women's fertility patterns change from no high-risk fertility behaviour to single to multiple high-risk fertility behaviour. It means women exposed to multiple high-risk fertility behaviour categories were highly prone to anaemia. In this study, any (either single or multiple) high-risk fertility behaviour independently pushes 16% of urban women in the reproductive age group to be anaemic. The findings of a previous study in Bangladesh also corroborate the notion that women of the reproductive age group who exhibit high-risk fertility behaviour were 12% (ARR = 1.12: 1.17–1.98) more likely to be anaemic [14]. In India, this turns out to be approximately 13.5 million urban Indian ever-married women (15–49 years) who could have been prevented from anaemia had they avoided high-risk fertility behaviour. Afterward, both single and multiple high-risk fertility behaviors were significantly associated with anemia, pushing 16% and 24% of urban women to develop anemia, respectively. A study from Ethiopia suggests that single and multiple high-risk fertility behaviour was highly significantly associated with anaemia among urban Ethiopian women [17]. The findings of this study regarding the control variables suggest that women who were using traditional methods (4 percentage points higher) of contraception were more likely to develop anemia than those who were using modern methods. This indicates that the use of traditional methods was associated with a higher likelihood of developing anemia among urban women. These results align with a study conducted in Maharashtra districts, India [28], which indicated a 10% higher chance of developing anaemia with the use of traditional contraceptive methods compared to modern methods. It could be so as women using traditional methods of contraceptives tended to have unplanned pregnancies and, hence more likely to have induced abortions or unplanned births, as the case may be. This could also have an adverse impact on women’s anaemic status as well. Similarly, a study from Eastern Africa found that the use of modern contraceptive methods was associated with a lower prevalence of anaemia [29]. Therefore, this study has a high program and policy recommendations.

The results on the association between anaemia and high-risk fertility behaviour reveal the state of women's health and well-being. According to the findings, these effects remained even after considering maternal characteristics, different socio-economic status categories, and demographic variables, all significant confounders of female fertility and anaemia. Urban, ever-married women's high-risk fertility behaviour is associated with anaemia, demonstrating how risky reproductive behaviour causes anaemia in these women. There are several alternative reasons for how high-risk fertility behaviour may raise the chance of anaemia in urban ever-married women. For instance, having children in short intervals prevents a mother from having enough time to recover from the physical strain of her first birth before commencing the following [19, 20].

Additionally, having too many and too frequent children puts the mother at risk for illnesses and other anaemia due to insufficient care [21, 22]. Anaemia is prevalent since early childbearing can cause significant blood loss during delivery [23]. Early motherhood and educational attainment might negatively impact women's anaemia levels. The age of marriage also tends to be higher when one's educational level is higher. As a result, raising women's education level can address the issue of early marriage and motherhood [24, 25]. Women with closely spaced pregnancies, a higher number of live births, or a trend of too-early or too-late childbearing are more likely to display certain behavioural risk factors, such as failure to use healthcare services and sociodemographic disadvantages [26]. Furthermore, one Indian study suggests that educational attainment and household wealth status were India's most significant factors in using maternal healthcare services [27]. Thus, these two determinants can affect women’s anaemic status indirectly too putting them in a double disadvantageous position. South Korean women over 35 years at the time of birth have a higher risk of early neonatal mortality [32], and a study based on China suggest that maternal anaemia was significantly associated with the advance maternal age (> 34 years) with higher odds ratio (AOR = 1.386) and neonatal complications [33], indicating a high-risk birth outcome and associated complications.

## Conclusion

In India, urban married women showed maternal high-risk fertility behaviour, likely impacting women's health. The study's findings reveal that more than half of these mothers were anaemic. Our research leads to a significant conclusion that the findings could help in reducing risk of anaemia among urban Indian women aged 15–49 by mending their fertility behaviour along with other community or public health interventions. The study's findings may also be applicable in areas where mothers of young children frequently suffer from anaemia. Policies and choice-based family planning methods should be used to reduce high-risk fertility behaviour among urban Indian women that would not only help to improve their own health and well-being but also the children born to them.

## Data Availability

The datasets generated and analyzed during the current study are publicly available on the DHS website one can collect the data. The data used in this study is available at request at https://www.dhsprogram.com.

## Abbreviations

WHO: World Health Organization
NFHS: National Family Health Survey
CAB: Clinical Anthropometric and Biomarker
HRFB: High-risk fertility behaviour
